# Process evaluation of a National Primary Eye Care Programme in Rwanda

**DOI:** 10.1186/s12913-018-3718-1

**Published:** 2018-12-07

**Authors:** Jennifer L. Y. Yip, Tess Bright, Sebastian Ford, Wanjiku Mathenge, Hannah Faal, on behalf of the Rwanda Primary Eye Care Process evaluation group, Theophile Dushime, Hannah Faal, Sebastian Ford, Wanjiku Mathenge, Marie-Aimee Muhimpundu, David Musendo, Eliana Ndererimana, John Nkurikiye, Vincent Tuzinde, Pacifique Uwamahoro, Abdallah Uwihoreye, Jennifer L. Y. Yip

**Affiliations:** 1 International Centre for Evidence on Disability, London School of Hygiene & Tropical Medicine, Keppel Street, London, WC1V UK; 2 International Centre for Eye Health, London School of Hygiene & Tropical Medicine, London, UK; 3 Vision for a Nation Foundation, London, UK; 4 Rwanda International Institute of Ophthalmology and Dr Agarwal’s Eye Hospital, Kigali, Rwanda; 5 Africa Vision Research Institute, Durban, South Africa, University of Calabar, Calabar, Nigeria

**Keywords:** [MeSH] primary health care, Delivery of health care, Integrated, Evaluation

## Abstract

**Background:**

Visual impairment is a global public health problem, with an estimated 285 million affected globally, of which 43% are due to refractive error. A lack of specialist eye care in low and middle-income countries indicates a new model of care would support a task-shifting model and address this urgent need. We describe the features and results of the process evaluation of a national primary eye care (PEC) programme in Rwanda.

**Methods:**

We used the Medical Research Council process evaluation framework to examine the implementation of the PEC programme, and to determine enablers and challenges to implementation. The process evaluation uses a mixed methods approach, drawing on results from several sources including a survey of 574 attendees at 50 PEC clinics, structured clinical observations of 30 PEC nurses, in-depth interviews with 19 key stakeholders, documentary review and a participatory process evaluation workshop with key stakeholders to review collated evidence and contextualize the results.

**Results:**

Structured clinical assessment indicated that the PEC provided is consistent with the PEC curriculum, with over 90% of the clinical examination processes conducted correctly. In 4 years, programme monitoring data showed that nearly a million PEC eye examinations had been conducted in every health centre in Rwanda, with 2707 nurses trained. The development of the eye health system was an important enabler in the implementation of PEC, where political support allowed key developments such as inclusion of eye-drops on the essential medicines list, the inclusion of PEC on insurance benefits, the integration of PEC indicators on the health management information systems and integration of the PEC curriculum into the general nursing school curriculum. Challenges included high turnover of primary care nurses, lack of clarity and communication on the future funding of the programme, competing priorities for the health sector and sustained supervision to assure quality of care.

**Conclusions:**

A model of a national primary eye care programme is presented, with service delivery to all areas in Rwanda. Key learning from this evaluation is the importance of strengthening the eye health care system, together with a strong focus on training primary care nurses using a PEC curriculum.

**Electronic supplementary material:**

The online version of this article (10.1186/s12913-018-3718-1) contains supplementary material, which is available to authorized users.

## Background

Globally, there are 36 million people blind and 400 million people with visual impairment. The Global Burden of Disease (GBD) study also estimates over 1 billion people worldwide are affected by presbyopia [1]. Though age-specific prevalence is decreasing, population ageing and growth results in a continued rise in numbers of people with poor vision [1]. There are inequalities in eye health, with the greatest burden of Vision Impairment (VI) borne by the poorest countries, which have the least resources to alleviate the impact of disability in their populations.

The World Health Organization’s (WHO) Global Action Plan 2014–2019, Universal Eye Health, steers a global response to reduce avoidable VI through improving access to comprehensive eye care services that are integrated into health systems [2].

Universal access to eye care is dependent on the development of eye health systems, where the health workforce is a key determinant of success. There is a severe shortage of eye care specialists in low-income countries, which limits national capacity to address VI. Task-shifting, a process of delegation where clinical tasks are shifted to less specialized health workers where appropriate, has been used successfully to improve access to HIV services in low income countries [3]. Adaptions of this strategy for cataract and trichiasis surgery have had limited impact [4, 5]. Training primary healthcare workers to deliver primary eye care (PEC) offers a pragmatic approach to address the eye health workforce shortages through improving access and reducing demand for specialist care. This strategy also aligns to WHO’s framework on integrated, people-centred health services, which re-orientates service delivery to patients and communities, that is accessible and of high quality [6]. However, there is limited evidence of the effectiveness of PEC [7].

Since the first Prevention of Blindness Plan was written in Rwanda in 2002, the Ministry of Health in Rwanda with support from international non-governmental organisations (iNGOs) has worked to develop a PEC programme for Rwanda. The organisation of eye care services and collaboration between stakeholders in Rwanda, under a single national plan that is regularly updated has been previously described [8]. A previous assessment of a former PEC programme in the Western Province raised concerns about the competency of general health workers in PEC [9, 10] and their ability to accurately identify and refer patients with eye complaints [11]. This learning led to the development of a national curriculum for PEC in Rwanda and a new programme between 2012 and 2013.

The development of the national PEC curriculum for Rwanda resulted in standardized training for general primary care nurses, competencies for delivery of PEC and management flow charts. PEC was not designed to detect eye diseases, but to provide a basic eye examination that differentiates normal eyes from abnormal eyes and skills for managing patients with minor eye conditions and referring more complex cases seen in primary health centres. The outcomes of PEC examination included counseling and education, diagnosis and treatment of minor eye conditions (including conjunctivitis and dry eye), diagnosis and treatment of uncorrected refractive error (URE) in adults and presbyopia with adjustable glasses or non-prescription reading glasses respectively, and referral to secondary care for more complex cases. An outline of the PEC management flow diagram is shown in Additional file 1: Figure S1.

The aim of this article is to describe the participatory process evaluation of the PEC programme in Rwanda from 2012, to identify key learning for policy and practice in delivery of PEC in a low-income setting.

## Methods

**Fig. 1:**
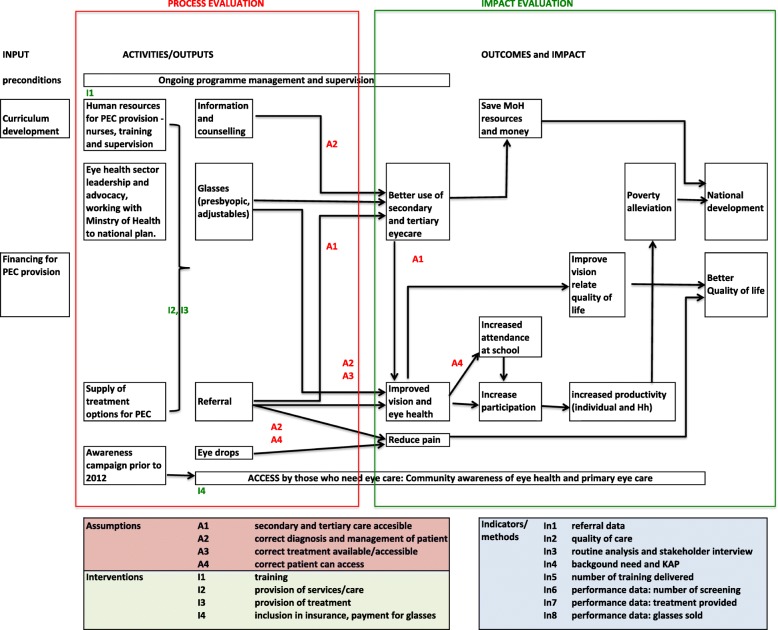
Theory of change and scope of process and impact evaluation for Primary eye care in Rwanda. Figure to illustrate theory of change

The process evaluation was set within a wider impact evaluation. The scope and considerations of each are shown in Fig. 1. In this article, the “PEC programme” refers to the PEC programme of activities launched in 2010 by the Rwandan government and supported by Fred Hollows Foundation (curriculum development) and Vision for a Nation Foundation (implementation) [8].

### Overview

The process evaluation uses a mixed methods approach, drawing on results from several sources including:

- survey of clinic attenders
- structured observations from an ophthalmic clinical officer
- in-depth interviews with key stakeholders
- programme and published documentary review
- participatory process evaluation workshop with key stakeholders to review the collated evidence and make recommendations for further development to ensure sustainability of the service.

We examined how successful the implementation of PEC was using the Medical Research Council (MRC) process evaluation framework [12], with consideration to the fidelity, dose, reach and adaptation of the PEC programme.

The stated goal or vision of the PEC programme was to provide nationwide access to eye care and affordable glasses for all. The initial intention, prior to the project consultation and design phases, was focused on provision of low cost adjustable glasses to underserved populations. These intentions rapidly evolved to improving access to eye care due to RMoH’s assessment of the need for broader eye health services at the primary level. VFAN worked with the Rwanda Ministry of Health Technical Working Group for Eye Health and other partners to design a programme that would integrate provision of glasses with PEC through the development of a national PEC curriculum and training a PEC workforce to deliver services.

Based on the description of activities and review of documents, we applied a health systems strengthening perspective to understand key enablers and challenges to implementation.

### Design phase

A workshop was held in Rwanda in May 2016 to develop a theory of change for the PEC programme. The integration of theory of change with the MRC process evaluation framework is a robust approach, enabling identification of gaps and areas for further research. This method has been previously described [13]. Individual clinical outcomes of PEC (outlined above) were identified with local stakeholders and linked to potential impact through a variety of pathways. Inputs and activities were identified from organization documents and stakeholder interviews. Any assumptions underpinning the various pathways were documented. Indicators for measuring the outcomes and impact were defined through literature review, and in discussion with stakeholders.

### Analytical framework

We analysed all described elements of collected data using an integration of the MRC process evaluation framework [12] and the theory of change (Fig. 1). Within the process evaluation framework, key considerations are contextual factors and implementation. Here, the intervention is PEC and components of its service delivery.

The contextual factors that affected implementation were considered within WHO health system building blocks and how these factors affected access to PEC health services [14]. We examined implementation of PEC with regard to fidelity, dose, adaptation and reach. The intervention can be considered the package of care delivered in PEC, as defined by the PEC curriculum. Therefore, the structured clinical observations, based on structures (equipment) and process (examination of patients) indicators can be considered as an investigation into how true the delivery of the intervention was compared to the intended intervention.

The source of data for each component of the analytical framework is shown in Table 1. For all elements of the framework, we used primary data, with triangulation from an alternative source as outlined in the table.

**Table 1:** Analytical framework and methodological source of data

Domain	Element or indicator for analysis	Source of data
Contextual Factors		
Leadership and governance	Engagement of Ministry of Health and key stakeholders for delivery of PEC	Stakeholder interviews Documents (Memorandum of understanding, published literature [9])
Healthcare Financing	Funding for PEC delivery, Funding for patient access to PEC	Stakeholder documents Rwanda Ministry of Health reports KAP survey
Health workforce	PEC nurses trained PEC nurse supervision	VFAN programme monitoring data Stakeholder interviews Structured observation interviews
Medical products, technologies	Availability of equipment (see Additional file 1: Figure S1) Availability of treatment options (antimicrobial and antihistamine eye drops, glasses)	Stakeholder interviews Structured observations RMOH reports VFAN programme documents and data
Information and research	Primary Eye Care routine monitoring data	RMoH reports VFAN programme documents and data
Service Delivery (see implementation below)	Number of PEC nurses per health facility and per population Number of PEC examinations delivered	VFAN programme documents and monitoring data
Implementation		
Fidelity	Number of nurses and OCOs trained	VFAN programme documents
	Adherence to curriculum	Structured observations
Dose	Number of PEC examinations delivered	VFAN programme monitoring data
	Number of glasses, eye drops prescribed and referrals made	VFAN programme monitoring data
Adaptation	Changes to PEC programme	VFAN programme documents Stakeholders interviews
Reach	Geographical spread of services Access by those who need PEC	VFAN programme monitoring data

*PEC* primary eye care, *RMoH* Rwanda Ministry of Health, *KAP* knowledge attitude and practice, *VFAN* Vision for a Nation

### Data collection

#### Clinic survey

A clinic survey was conducted as part of a national survey of background need for PEC. A nationally representative sample was selected through a two stage sampling process using probability proportional to size approach, with the national census data as the sampling frame. The two stages selected 10 districts and subsequently 5 villages from each district, resulting in 50 villages. We surveyed attendees at the PEC clinic that served each of the 50 villages to determine their satisfaction with the services received. We asked the attendees the following question: *“On a scale of 0 to 10, how likely are you to recommend PEC to a friend or a colleague?”* The scores were categorised into 0–6 for poor, 7–8 and 9–10 high satisfaction.

#### Stakeholder interviews

Semi-structured in-depth interviews were conducted in English by an experienced researcher or in Kinyarwanda by a trained local fieldworker. Topic guides were developed, and covered a range of themes including: role of the interviewee and their connection to VFAN, aspects of the primary eye care programme, implementation of the programme (including training and supervision), benefits and shortcomings of the programme. The questions were adjusted according to the role and experience of the stakeholder interviewed. Nineteen interviews were conducted. The interviews took place in the stakeholders’ workplace in Rwanda or in London. We used purposive sampling to ensure clinicians involved in eye care were included, in addition to decision makers and members of the technical advisory group for PEC in Rwanda. The range of stakeholders included in the study is shown in Table 2. Qualitative data were analysed by a qualitative researcher using the framework approach. This involves developing an analytical framework from the interview data and charting data into a matrix developed using Excel to cross reference themes and data.

**Table 2:** Role and numbers of stakeholders interviewed

Role	Number of interviewees
Implementers	
Ophthalmic Clinical Officers	3
Primary eye care nurses	4
Health centre manager	1
Vision for a Nation personnel	
Country Director	1
Former CEO	1
OCO training manager	1
Director of Partnerships	1
Founder	1
Other key stakeholders	
Director General of Clinical services and Public Health (Ministry of Health)	1
Senior Rwandan Ophthalmologists	2
Fred Hollows Country Director	1
One Sight Country Director	1
Ophthalmic Clinical Officer, One Sight, University of Rwanda	1
Total	19

#### Structured observations

A structured observation based on the PEC curriculum was conducted by a Rwandan Ophthalmic Clinical Officer (OCO), who was also a PEC trainer and therefore familiar with the clinical flow charts and competence requirements of PEC nurses. We used the PEC manual and curriculum to elicit structure and process [15] indicators of the PEC consultation and recorded into a mobile data collection instrument (Additional file 1: Table S1). The OCO observed each PEC nurse examinations for one clinic session. The assessment was made on the first two patients, allowing time for guidance and supervision where required. We sampled thirty nurses working in fifteen different health centres through purposive sampling, across all five provinces in Rwanda, and nurses observed with consent in their usual workplace. After the structured observations, we also interviewed the nurses using a questionnaire to determine their experience and views on training and supervision, and job satisfaction.

### Document review

Key programme documents from Vision for a Nation were reviewed and are listed in Additional file 1: Table S2. The main programme document was produced retrospectively. We also reviewed available documents from the Rwanda Ministry of Health [16] and published literature to provide context and background to the development and delivery of PEC in Rwanda [8–10, 17]. The programme office also supplied an overview of the programme monitoring data and referral data.

### Participatory process evaluation workshop

The process evaluation data was reviewed by an independent expert (HF). Following this, findings were presented at a process evaluation workshop held in Kigali. The aims of the workshop were to: corroborate data, agree interpretation of key findings, identify areas of good practice, identify challenges, and determine next steps for further development of PEC in Rwanda. This workshop included a site visit to a health centre delivering PEC, further stakeholder interviews and focus group discussions. Attendees participating in the workshop included key representatives from the Ministry of Health (TD), the Rwanda Biomedical Centre (MM), an Ophthalmologist and Professor at University of Rwanda (CM), Country Director for VFAN (AU), Fred Hollows Foundation (EN), OneSight (VT), Director of Strategy for VFAN (SF), independent consultant with previous work for VFAN (DM), researcher and chaired by the independent expert (HF). Those who were unable to attend in person provided written feedback and input.

### Analysis

Data analysis from all methods outlined above combined inductive and deductive approaches, with themes drawn from the theory of change and analytical framework and emerging from empirical data, subsequently tested with data sources. Findings were considered with the workshop participants and triangulated with other sources of collected data for reliability and validity.

### Ethics, consent and permissions

This study was approved by the Rwanda National Ethics Committee (725/RNEC/2016) and the ethics committee of the London School of Hygiene &Tropical Medicine. All participants, or their legal guardians, provided informed written consent to participate in the study or to publish individual data.

## Results

### Context

An overview of contextual factors that influenced implementation of the national PEC programme is shown in Table 3. The health system in Rwanda is organized around local health centres, each serving a population of between 4000 and 10,000 people formed a basis for the delivery of PEC. Engagement and support from the Rwanda Ministry of Health (RMoH) to the eye health sector(joint paper published [8] and Memorandum of understanding) provided the political leadership and influence to enable establishment of PEC activities. One stakeholder describes this:

*“Integral to the whole programme is developing it in full partnership with the Ministry of Health and it being the Ministry’s programme with us providing the support to the Ministry. Obviously that is critical to having a nationally owned and sustainable programme.” [Stakeholder 013]*

**Table 3:** Enabling factors and challenges for sustainable Primary Eye Care programme implementation in Rwanda

Health system domain	Enabling Factors	Challenges
Leadership and Governance	Commitment and support to PEC programme by Ministry of Health, with memorandum of understanding in place for delivery of PEC Good engagement between VFAN and RMoH, and other eye sector stakeholders	The eye health technical working group can be strengthened
Healthcare Financing	PEC examination included in community health insurance coverage External funding raised to support PEC and outreach activities	Cost of glasses can remain prohibitive for the poorest Payments received for glasses are held centrally rather than in health centres with no clear communication of plans for allocation
Health workforce	Integration of PEC curriculum into nursing schools	Turnover of PEC trained health centre nurses leaving gaps in provision of PEC clinics. Supervision can be strengthened, with reports of inconsistency in frequency and purpose. Competing priorities at health centres limits availability of nurses to provide PEC
Medical products and technologies	Inclusion of eye drops on essential medications list Secured supply chain for non-prescription reading glasses and adjustable glasses.	Variable availability of eye drops and glasses can limit management options
Information and research	Integration of PEC indicators on health management information systems data, co-ordinated by RMoH.	Data is held centrally and access can be difficult. Primary care data not routinely linked to secondary or tertiary care data, but collected locally by VFAN.
Service Delivery	Successful integration of PEC delivery into health centres	Interface between different levels of care could be strengthened – increase communication and feedback between primary and other levels of care.

The agreement between RMoH and VFAN was expressed through memoranda of understanding from 2012 to 2015, and from 2015 until end of 2017, where the key change in service delivery was inclusion of outreach activities to villages. RMoH support and advocacy from eye care partners was a key factor in driving other enabling contextual factors such as inclusion of PEC in community insurance reimbursement (*Mutuelle de Sante*), inclusion of antimicrobial eye drops on the list of essential medicines, and inclusion of PEC indicators on the health management information system (HMIS).Though there is continued support from RMoH, eye care sits within the non-communicable disease (NCD) division, where there are competing priorities with high profile conditions such as diabetes and cancer. The RMoH annual reports do not consistently profile eye health and there is no statement of prioritization for eye care, in contrast to other NCD areas. Raising awareness of eye health is no longer part of community health workers’ remit (documents 1 and 9). Stakeholders also stated that the MOH technical working group for eye health, which is convened by the Rwanda Biomedical Centre (RBC, the delivery arm of the RMOH), could be strengthened, with more frequent meetings and updates, and RBC taking a leadership role for the eye-care sector.

The financial investment from external donors prior to establishment of the service was also an important input in the framework. These funds provided the pump priming finance required to implement a national PEC programme, through curriculum and workforce development. The PEC programme also developed a sustainable supply chain for provision of glasses, with payments from patients pooled into an MoH revolving fund allocated for financing future PEC and eye care service delivery. Though the memorandum of understanding between RMoH and VFAN outlines the commitment to allocate healthcare staff and resources to support delivery of PEC, there was a lack of clarity amongst stakeholders on the financial commitment from the RMoH, and the status of the RMoH revolving fund. VFAN also provided the stockpile of glasses, which can sustain the services in the near future, though it is unclear on how the funds will be allocated.

The current PEC programme incorporated learning from previous PEC experience in Rwanda in order to improve outcomes. This included the establishment of the national PEC curriculum, a key foundation to improving the quality of care delivered. The new programme curriculum provided a set of clinical care protocols, appropriate for the skillset of primary care nurses, which increased consistency of services delivered. It also provided a clear structure for competency-based training of the PEC workforce.

Training and supervision of a skilled PEC workforce is an important component of strengthening the eye-care system. Prior to the current programme, there were few general health workers trained in PEC and limited capacity of specialist services. The current programme has trained over 2700 nurses, with at least two trained PEC nurses employed in each health-centre.

The clinical OCO assessment took place between February 2017 and June 2017, where we assessed and interviewed 30 nurses in nineteen different health centres. This was higher than the expected 15 health centres as only one nurse was available on the day of the assessment in some health centres where at least two were expected.

The structured assessment indicates that the PEC provided is consistent with the curriculum, with over 90% of the clinical examination processes conducted correctly (Table 4). This also suggests that the training received was effective in developing nurses’ skills to deliver consistent PEC care. A majority of the nurses (87%) were satisfied or very satisfied with their work. PEC nurses indicated that more regular training and supervision would be welcomed. The average time between training and refresher courses was 2.5 years, with 90% indicating they would like more frequent training. Supervision is provided by OCOs as part of their contractual duties with the district hospitals, though the frequency and content of the supervision varies. The issue of training and supervision are closely linked, and greater frequency of supervision based learning and development could reduce the need for formal training. One interviewee stated:

*“We are just waiting. They told us that we would get supervisors from district hospital, but until now we have not seen any.”[Stakeholder code 003]*

**Table 4:** Process elements of structured clinical observations on 30 primary eye care (PEC) nurses in their usual workplace

Observation element	Percent
Structures/Equipment	
Eye record book and referral forms	76.7
Eye examination protocol/checklist (proforma for examination)	43.3
Rope (to measure correct distance for VA testing)	83.3
Visual acuity chart	96.7
String (to measure correct distance for reading vision test)	76.7
Reading chart	93.3
Pinhole	93.3
Torch	70.0
Reading glasses (for treatment)	100
Adjustable glasses	93.3
Eye dressings	6.7
Eye pad and cotton buds	3.3
Tape	80.0
Gloves	10.0
Antibiotic eyedrops	93.3
Anti-allergy eye drops	73.3
Flowchart (management algorithm)	66.7
Processes	
History taken in accordance to PEC curriculum	93.3
Eye examination	
Good communication and explanation prior to examination/test	93.3
Observations of eyes made	96.7
Distance visual acuity test procedure explained	86.7
Effective communication with patient	93.3
Correct distance applied for distance VA	100
Appropriate lighting conditions for distance VA test	93.3
One eye covered well for distance VA test	93.3
Correct VA recorded for distance test	90.0
Pinhole test offered for correct patient	90.0
VA related diagnosis correct	96.7
Explanation of near VA test offered	90.0
Effective communication for near VA test	95.2
Correct distance for near VA test	93.3
Appropriate lighting conditions for near VA test	100
Correct VA recorded for near test	96.7
Correct diagnosis for near test	96.7
Management	
Correct management plan	96.7
Correct glasses offered	100.0
Correct eyedrops offered (*n* = 13)	84.6
Instructions provided with correct eyedrops (n = 13)	69.2
Consultations resulting in significant error (examination results, diagnosis or management of patient)	6.7

*VA* visual acuity

The high turnover of PEC trained health-centre nurses posed an initial challenge to service delivery. However, this led to increasing the number of trained nurses and the integration of PEC into the Rwandan nursing schools training curriculum, resulting in a mechanism for a continual supply of PEC trained nurses. As new cohorts of nurses enter the workforce, they may need additional support to raise confidence as they gain experience in PEC. Additional quality assurance through comparisons with current in-service training is required. On site training using peer networks with OCO supportive supervision can also have a role in continued quality assurance of PEC.

The current service is delivered at health centres as a separate PEC clinic, at a specified time during the week, which is communicated to the patients. This allows for PEC nurses to consolidate their skills in focused sessions, rather than eye examinations dispersed amongst a range of other clinical examinations. Competing priorities in other areas of healthcare such as maternal health and infectious diseases can limit the capacity of trained nurses to deliver PEC clinics. However, between 2015 and 2017, the provision was augmented with outreach activities, where nurses were paid to deliver PEC services in villages within the health centre’s catchment area. Additional funding was sought from international aid grants to deliver two outreach PEC clinics in all 15,000 villages in Rwanda. This resulted in a significant increase in the number of examinations delivered (Table 5). As the outreach was funded by external donors, it is time limited and intended to raise awareness and reduce the backlog in need for PEC. This increase in demand also built the level of experience amongst PEC nurses. However, some stakeholders raised concerns this may mislead service users about where to seek eye care in the future. (see section on Adaptation). Eye-drops were only available for people attending clinics and not those attending PEC at outreach, where only prescriptions were available. Referrals were made through a paper-based system, and patients were instructed to attend a referral hospital with a form. There were no records of outcomes of referrals in the primary care patient record book. The outcomes of referrals are collected by district hospitals, with paper-based feedback to a central office in the health centre, but were not part of patient records.

**Table 5:** Outputs from primary eye care (PEC) programme

	November 2012–September 2015 Before outreach	September 2015–October 2016 After outreach	Total
Number of PEC eye examinations	352,830	619,465	972,295
Refractive errors diagnosed	54,709	84,648	139,357
Referrals made	38,657	63,734	102,391
Number of villages reached through outreach (total = 15,000)		11,487	11,487
Number of OCOs trained as PEC trainers			32
Number of nurses trained			2707

*OCO* ophthalmic clinical officers

### Implementation

#### Fidelity

The results of the structured clinical observation showed that a majority, though not all, of nurses reviewed had the appropriate equipment to deliver eye examinations. However, less than half of the clinics observed had materials to treat eye injuries, such as eye dressings and eye pads. In stakeholder interviews, there were some reports of difficulties with the supply of eye-drops for treatment of allergic conjunctivitis, which was supported by the clinical observations. 73% of clinics stocked anti-allergy eye drops compared to 93% for antibiotic eye-drops in the clinical observations. However, as the medications were part of the essential medications list, it was considered the health-centres’ responsibility to ensure availability. Equipment that could reduce human factors in delivery of PEC such as the examination check-lists and management flowchart were only present in 43 and 67% of clinics respectively. Overall, the delivery of eye examination and management plan were considered acceptable for over 90% of the the clinical examinations observed.

#### Dose

Outputs from the PEC programme since 2012 are shown in Table 5. Between 2012 until 2015, over 350,000 eye examinations were conducted in PEC, with over 50,000 refractive errors diagnosed and over 38,000 referrals made to secondary care. From 2015 until October 2016, there was a significant increase in the number of eye examinations, in part due to outreach clinics (see Table 5). From 2012 to October 2016, 32 OCOs have been trained to deliver PEC training, and 2707 PEC nurses have been trained, with at least 2 PEC trained nurses employed at each health centre.

#### Adaptation

The PEC curriculum was designed for delivery at primary care and health centres. In 2015, the time limited outreach programme was proposed and initiated with external funding. The driver for this change in PEC delivery was to increase uptake and raise awareness of PEC. There are mixed views on the impact of outreach PEC as demonstrated from interviews from these stakeholders.

*“The outreach programme is a good thing on a short term. It is not something you can sustain. The nurses use the afternoons to go to villages to do exactly what they do in health facilities. So that It adds value because, first of all the people will know the health centres have workers who can do that job, and secondly it gives access to those who could not have come to the health centre for different reasons. But it is expensive.” [Stakeholder 006]*

*“The outreach programme is a good programme because nurses are going to the villages…. [however], there are some challenges. Nurses have many tasks at the health centre level and in outreach they have to work extra time.” [Stakeholder 008]*

As a consequence of the outreach, the numbers of eye examinations significantly increased (Table 5), allowing PEC to reach proposed targets outlined in the memorandum of understanding with the MoH. PEC nurses were not able to provide eye drops as treatment in outreach as this required a prescription dispensed at the health-centre. Delivery of outreach required additional payments for travel and nurses’ time, which made cost per examination more expensive. Some stakeholders also stated this raised expectations from PEC nurses on payment for PEC services.

*“My reservations are based on the fact that – how often can it happen and what happens when there are no allowances for those nurses to go to the community. They go because they have an allowance and this is the bit I don’t like. Because my thinking is that when they go to the community, they go in office hours, they are being paid a salary anyway. And if it had been just transferring your work from the station to the community – that I find sustainable. But when you link such a thing to an allowance, the minute that allowance stops, you know they see it now as a project, not as part of their daily work.” [Stakeholder 010]*

There were also concerns that provision of PEC in villages would change attitudes to PEC and patients would not travel to health centres for further access.

*“When you do start doing that, does it discourage the community from going to the health centre because they know they could just wait in their house and they will come to them. Which again I think is working against the sustainability of this because primary eye care was about developing the service in the static facility, not about, … it sort of became murky between primary eye care and community eye care at that point to me” [Stakeholder 010]*

#### Reach

Until June 2017, based on available data at the time of the participatory process evaluation workshop, over 11,000 villages had received outreach (out of 15,000) with estimated coverage to reach all villages by the end of 2017. This indicates that all areas of Rwanda will have received PEC outreach.

### Patient satisfaction

We surveyed 574 attendees at the 50 PEC clinics, of which 21% were patients at health-centres and 78% at village outreach clinics. Of these, 49.3% (95%CI = 45.1–53.5%) reported high levels of satisfaction with the service, with 24.4% (95%CI = 17.2–23.9%) reporting low levels of satisfaction. There was no association with high levels of satisfaction with age or sex. People who attended outreach were nearly twice as likely to report high satisfaction compared to those attending at health-centres (Odds ratio (OR) = 1.97, 95%CI = 1.25–3.10).

## Discussion

Integrated primary eye care re-orientates the focus of eye care activity from hospitals to local health centres, and prioritises primary care in the prevention of blindness, in line with the WHO framework on integrated, people-centred health services [6]. We have presented a model of eye-care that has delivered over a million PEC examinations to the Rwandan population through integration of primary eye care into the primary health care system.

This process evaluation has reported that the current programme has trained 2707 nurses (up to October 2016), and integration into the nursing curriculum will likely provide a sustainable workforce though further consideration to the structure and processes of the OCO supervision is required. Though the nurses had already provided PEC in principle, previous studies have shown that the levels of knowledge in PEC was low [9, 10]. Therefore raising quality of care required a revised curriculum, additional training and a period of horizontal *disintegration,* with PEC provided in separate clinics and outreach. With the inclusion of this revised PEC curriculum into the general nurses’ training, this will likely result in continued supply of PEC nurses that could again, provide PEC as part of their routine clinics. Both OCOs and ophthalmologists play a crucial role in building the capacity of PEC nurses, through supervision and feedback to ensure quality of eye care delivered. The varied reports of supervision provided, together with the important role of OCOs in the future development of PEC indicates that a standard model of OCO supervision for PEC nurses will be beneficial for continued quality improvement. A comprehensive and integrated reconfiguration of eye healthcare teams is required for long-term success of PEC as a task-shifting strategy [18]. Regular input from OCOs on PEC patients care, and similarly, input from ophthalmologists into OCO’s clinical work can develop into virtual teams and vertical integration across levels of care. Though this model will be limited by a shortage of OCOs and ophthalmologists, additional peer networks can also provide supportive supervision. In Rwanda, an on-site nurse-led mentorship for rural health centre nurses has been trialed with promising initial results on quality of care [19].

The PEC service is now well established and integrated into local health-centres. The results also show that PEC can be delivered consistently at primary care level, with large number of eye examinations undertaken as demonstrated by the programme monitoring metrics. However, integration of PEC with community care and secondary and tertiary care was less evident**.** There was no direct record of referral tracing observed in the primary care records, which limits continuity of care. A clear referral system and pathways will enable improved co-ordination of care for all patients. This will require harmonized processes between all health-centres and hospitals, allowing information to flow. This will also facilitate the development of integrated working between PEC nurses, OCOs and ophthalmologists and greater focus on integrated patient-centred health services.

At the community level, community health workers currently do not have a role in promoting eye health and care. Raising awareness of eye health in communities has a role in engaging and empowering patients and those who need eye care to access eye health services, and reduce inequities in access [6]. Previous research has shown that community health worker education in combination with health systems strengthening or community development activities has a positive impact on care-seeking and vaccination uptake in children [20]. However, as a standalone intervention the impact is limited. The impact of outreach on access is shown in Table 5, where 352,830 examinations were made over 3 years before outreach, compared to 619, 465 in ones year after outreach to 11,487 villages. The temporary outreach PEC services improved access, particularly for vulnerable people who have difficulties travelling, but it is not sustainable and its cost-effectiveness is not clear and will require further investigation. Additionally, eyedrops are prescribed in outreach, but dispensed in health-centres; this will likely reduce access for those living in more rural areas, and further investigation on the impact of this service limitation is also required.

Key enablers in the development of PEC, include support from RMoH, and continued advocacy from the sector, therefore raising the profile of eye care. The financial investment from external donors and strengthening of the supply chain for PEC treatments were also key to implementation. Additional enablers and success factors are shown in Table 3 and include inclusion of PEC examinations in community insurance reimbursement, eye drops in the essential medicines list and PEC indicators in HMIS. However, long-term success for any task-shifting strategy requires political and financial commitment [18]. This evaluation has shown that though there is political commitment, eye care sits within the NCD department, with competing priorities and conditions with greater population healthcare needs. There was a lack of universal understanding of the financial commitment for PEC other than the funds arising from external donors. The RMoH revolving fund is centrally managed and intended to fund a sustainable PEC service, however there is no clear communication to all stakeholders of its intended purpose leading to uncertainty on the future financial sustainability of PEC. Though leadership and support from RMoH can be maintained, the external funding is unlikely to continue at the levels in the years reviewed. The continuation of the PEC service will also require local clinicians and service providers to take leadership of the service with political support and guidance from RMoH. A multistakeholder forum, such as the technical working group and a well-communicated strategic approach can help to sustain the PEC service in the future.

There are limitations to this study. All programme metrics were collected internally, and could not be validated against HMIS data, due to differences in indicators collected and timeframes. We only collected data on 30 nurses due to resource limitations, and though they were sampled from across the country, the relatively small sample could introduce information bias. Observations by a clinician may also heighten nurse performance, and therefore the results could indicate higher quality of care than that provided in usual conditions. However, we triangulated findings made from observations with stakeholder interviews, which would reduce the potential impact of this bias.

## Conclusion

This study has described an integrated PEC model in Rwanda, leading to 2707 nurses trained and PEC provided in every health centre in the country. We have described contextual factors that have enabled implementation and challenges in the development and sustainability of the service. There has been significant progress in the strengthening of the eye health system and integration of PEC in Rwanda. The next important step that will be considered in the concurrent impact evaluation is the impact of PEC on eye health outcomes, including equity of access, and the impact of treating and correcting URE and presbyopia on an individual’s life.

## Additional file

Additional file 1: **Figure S1.** The Primary Eye Care Management Flowchart. **Table S1.** Indicators used in evaluation of PEC consultation. **Table S2.** Documents reviewed. (DOCX 93 kb)
