# Supplementary material for: Process evaluation of a National Primary Eye Care Programme in Rwanda
Source: BMC Health Serv Res. 2018 Dec 7;18:950. doi: 10.1186/s12913-018-3718-1 (PMC6286556; doi:10.1186/s12913-018-3718-1)
Supplement: Supplementary file 1 — Figure S1. The Primary Eye Care Management Flowchart. Table S1. Indicators used in evaluation of PEC consultation. Table S2. Documents reviewed. (DOCX 93 kb) [file 12913_2018_3718_MOESM1_ESM.docx]

**Figure S1. The Primary Eye Care Management Flowchart**

**
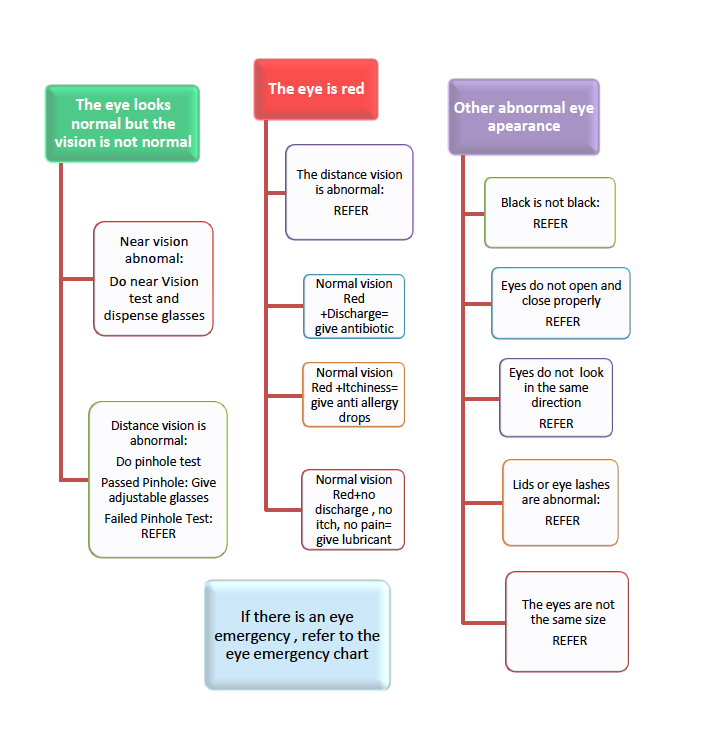
**

**Table S1. Indicators used in evaluation of PEC consultation**

| **EQUIPMENT LIST** |
| --- |
| A measure for 3m for distance visual acuity |
| A visual acuity chart that is in good working order |
| A measure for 40cm (near VA recording) |
| A reading chart in good working order |
| A pinhole |
| A working torch |
| Reading glasses of different prescriptions available for patients to try |
| Adjustable glasses for the patients to try |
| Antibiotic eye drops |
| Cromolin (for allergic conjunctivitis) eye drops |
| PEC training manual |
| Treatment algorithms |
|  |
| **OBSERVED EXAMINATION** |
| Did the nurse take a history in accordance to the curriculum?  - asking questions in the guidelines |
| Did the nurse explain the examination to the patient before starting? |
| Did the nurse look at the eyes? |
|  |
| **Distance VA testing:** |
| Did the nurse explain the test? |
| Is communication of the test effective?   - correct tone - words easily understood by patients – no jargon - speaks clearly - answers questions asked |
| Is the distance correct? |
| Is the light correct? |
| IS one eye covered properly for visual acuity in each eye? |
| Is the correct VA recorded |
| Is pinhole offered appropriately |
| Is the visual acuity related diagnosis correct  (good vision, URE, poor vision for referral) |
|  |
| **Reading Vision test** |
| Did the nurse explain the test? |
| Is communication of the test effective?   - correct tone, - words easily understood by patients – no jargon - speaks clearly - answers questions asked |
| Is the distance correct? |
| Is the light correct? |
| Is the correct VA recorded |
|  |
| **Management plan** |
| Is the diagnosis correct (see curriculum for what PEC nurses can diagnose) |
| Is the management plan correct  - the nurse should follow the curriculum algorithms |
| Did the nurse offer **correct** glasses? |
| Did the nurse offer **correct** eyedrops? |
| If nurses offered eyedrops – were the instructions on how to use them correct? |
| Did the nurse make an error?  ***If the nurse has made an error of diagnosis– please advise***  **Large –** incorrect VA, incorrect diagnosis and treatment (but not one that will lead to vision loss)  **Dangerous –** incorrect diagnosis that could lead to harm or vision loss) |

**Table S2. Documents reviewed**

| 1. Vision for a Nation programme design document |
| --- |
| 2. Primary Eye care training curriculum and manual  (developed by Ministry of Health and Wanjiku Mathenge) |
| 3. Memorandum of understanding between VFAN and Rwanda Ministry of Health |
| 4. VFAN application to UK Department of International Development Global Poverty action fund |
| 5. VFAN application to UBS Optimus |
| 6. VFAN application to USAID |
| 7. Previous independent evaluations commissioned by VFAN |
| 8. VFAN routine performance monitoring updates and data |
| 9. Rwanda Ministry of Health Annual Reports 2010-2011, 2011-2012, 2012-2013, 2013-2014, 2014-2015, 2015-2016 |
